# Alleviation of Brain Hypoperfusion after Preventative Treatment with Lomerizine in an Elderly Migraineur with Aura

**DOI:** 10.1155/2011/782758

**Published:** 2010-12-27

**Authors:** Joe Aoyagi, Ken Ikeda, Tetsuhito Kiyozuka, Takehisa Hirayama, Yuichi Ishikawa, Ryuta Sato, Yasuhiro Yoshii, Kiyokazu Kawabe, Yasuo Iwasaki

**Affiliations:** ^1^Department of Neurology, Mishuku Hospital, 5-33-12 Kamimeguro, Meguroku, Tokyo 153-0051, Japan; ^2^Department of Neurology, Toho University Omori Medical Center, 6-11-1 Omorinishi, Otaku, Tokyo 143-8541, Japan

## Abstract

Previous studies of brain single-photon emission tomography (SPECT) showed changes of regional cerebral blood flow (rCBF) in migraineurs during prodromes or headache attacks. Little is known about how successful medication of migraine prevention can reflect rCBF in migraineurs. We highlighted alternation of brain SPECT findings in a migraineur with aura before and after prophylactic treatment with lomerizine, a calcium channel blocker. A 70-year-old man with migraine developed visual disturbance frequently at walking exercise for the recent 3 months. After this visual attack, a mild-degree of throbbing headache occured occasionally. Brain SPECT using ^99m^Tc-ethyl cysteinate dimer was performed at interictal time of migraine. Brain SPECT before lomerizine treatment revealed hypoperfusion in the frontal, parietal, and occipital regions. He was diagnosed with recurrence of migraine with aura (MA). Lomerizine (10 mg/day, po) was administered for 3 months. MA and visual aura without headache were dramatically improved. Migraine attacks and visual disturbance were not induced at exercise. At 3 months after lomerizine medication, brain SPECT showed remarkable increase of rCBF. These SPECT changes of our patient indicated that antimigraine mechanism of lomerizine could contribute to restoration of cerebral hypoperfusion.

## 1. Introduction

Regional cerebral blood flow (rCBF) studies have been employed to investigate the pathophysiology of migraine headache [1–3]. The introduction of brain single-photon emission computed tomography (SPECT) using technetium Tc 99m- (^99m^Tc-) labelled hexamethylpropyleneamine oxime (HMPAO) or ethyl cysteinate dimer (ECD) has facilitated assessment of rCBF. A number of rCBF studies using Tc 99m HMPAO or ECD SPECT have been reported in patients with migraine during prodromes or headache phases [3–8]. However, little is known about the relationship between migraine preventative medication and rCBF. Lomerizine, 1-(bis(4-fluorophenyl)methyl)-4-(2,3,4-trimethoxybenzyl) piperazine dihydrochloride, is a calcium channel blocker with antimigraine properties [9]. This preventative drug is often used in Japan [10–13]. We first report a unique case in which prophylactic treatment with lomerizine recovered brain hypoperfusion on ^99m^Tc ECD SPECT during interictal period, in addition to dramatic amelioration of migraine attacks.

## 2. Case Report

A 70-year-old man developed visual disturbance frequently at walking exercise for the recent 3 months. Visual disturbance consisted of scintillating scotoma in both eyes, which continued 5–20 minutes. After this visual attack, a mild-degree of throbbing headache occured occasionally. He had prior history of migraine with aura (MA) and without aura (MO) from 30 years of age. After 60 years of age, migraine attacks were decreased to a few times per one year. Physical and neurological examination was normal during interictal periods. Neuro-ophthalmic examination was normal. Routine laboratory tests suggested mild degree of diabetes mellitus. Fasting blood sugar level was 144 mg/dL, and hemoglobin A1c was 6.6% (normal 4.3–5.8). Cerebrospinal fluid study was normal. Brain magnetic resonance imaging and angiography were not remarkable. Electroencephalogram was normal.

### 2.1. Measurement and Analyses of rCBF

Brain SPECT scanning with ^99m^Tc-ECD was performed at the interictal time of migraine. SPECT was scanned at 10 minutes after intravenous bolus injection of 1.5 mL (600 MBq), ^99m^Tc-ECD SPECT examination was performed using a rotating *γ*-camera (Prism 3000; Picker Corp. USA). Brain SPECT data were analyzed by the following two methods. One of SPECT analyses used the revised version of 3-dimensional stereotaxic region of interest template (3DSRT) by Takeuchi et al. [14]. A total of 636 ROIs were set in bilateral cerebral cortexes and cerebellar hemispheres. Global CBF was calculated from all data of 636 ROIs in whole brain, including both cerebral hemispheres and cerebellum. SPECT images were divided as regional CBF into 24 symmetrical (right and left) regions per patient: the callosomarginal, the precentral, the central region, the parietal region, the angular region, the temporal region, the posterior region, the pericallosal region, the lenticular nucleus, the thalamus, the hippocampus region and the cerebellar hemisphere. Quantification of rCBF was assessed using the noninvasive Patlak plot method without blood sampling [15]. Data of global and regional CBFs were shown in mL/100 g/min.

Another method was analyzed by easy Z-score imaging system (eZIS).

### 2.2. rCBF Alternation before and after Lomerizine Administration

**Figure 1:**
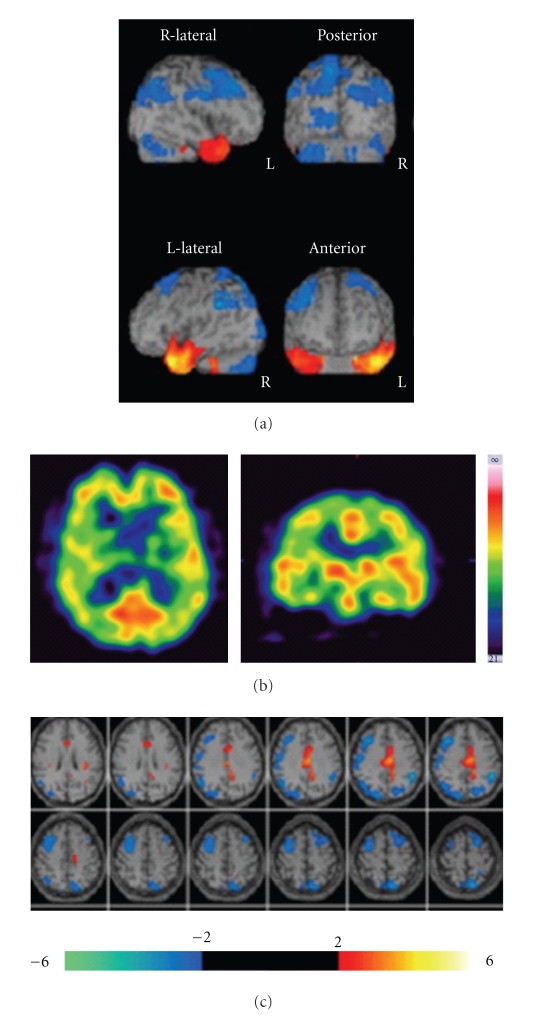
Brain ^99m^Tc-ECD SPECT imaging before lomerizine treatment, CBF is decreased in bilateral frontoparietal and the left occipital regions on eZIS imaging.

**Figure 2:**
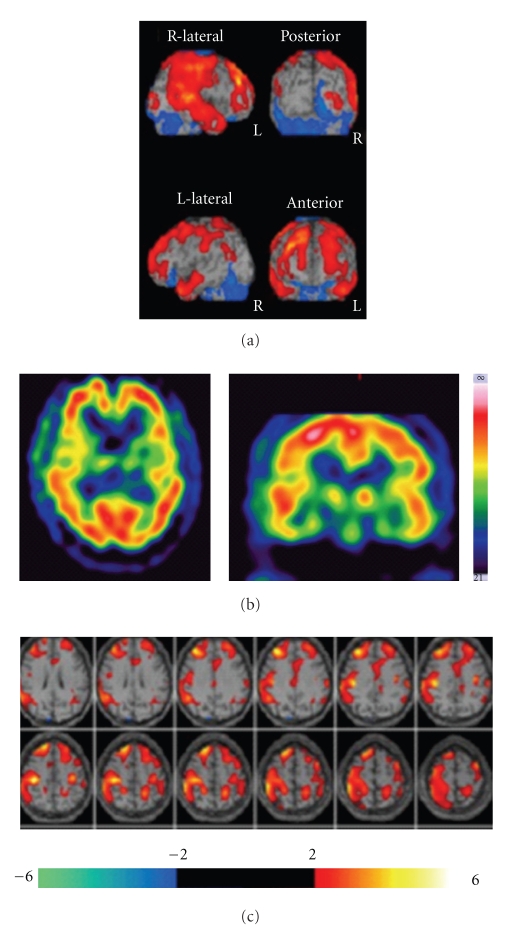
Brain ^99m^Tc-ECD SPECT imaging after lomerizine treatment. As compared to pretreatment with lomerizine, CBF is increased in the bilateral frontoparietal regions on eZIS imaging. Cerebral hypoperfusion is improved markedly.

Brain ^99m^Tc-ECD SPECT before lomerizine treatment revealed hypoperfusion in the frontal, temporal, parietal, and occipital lobes (Table 1). Reduction of rCBF was detected predominantly in the right hemisphere (Figure 1). He was diagnosed with recurrence of MA. Lomerizine (10 mg/day, po) was administered for 3 months. MA or visual aura without headache was dramatically improved. There were no migraine attacks and visual disturbance whenever he walked and exercised for long time. The second ^99m^Tc-ECD SPECT was performed. As compared to pretreatment with lomerizine, rCBF was increased in most of the cerebral cortex (Table 1). Restoration of frontoparietal hypoperfusion was found on eZIS imaging (Figure 2).

## 3. Discussion

We showed that prophylactic treatment with lomerizine ameliorated brain hypoperfusion during the interictal period in a patient with MA, together with complete prevention of migraine attacks and visual auras.

Lomerizine, an antimigraine calcium channel blocker, is prescribed widely in Japanese migraineurs [10–13]. Effectiveness of this prophylactic medication is approximately 50%. Lomerizine belongs to the same class of diphenylpiperazine-type calcium antagonists as flunarizine [9], and this drug is prescribed for migraine prophylaxis in Japan. Previous clinical trials of lomerizine suggested that this drug reduced the frequency of migraine attacks over 12 weeks [10–12]. Propranolol, amitriptyline, and valproate sodium are used internationally as preventative medication. Little is known about how these drugs influence rCBF on brain SPECT during the headache attack or the interictal phase in migraineurs. Prophylactic effects of magnesium citrate supplementation (600 mg/day, po) were assessed by means of clinical evaluation, visual evoked potential, and statistical parametric mapping of brain SPECT before and after 3 months treatment. Magnesium treatment significantly increased CBF in the inferolateral frontal, inferolateral temporal, and insular regions [16]. Previous studies disclosed antimigraine effects of lomerizine in animal models [17–20]. Inhibitory effects of lomerizine on the cortical hypoperfusion and expression of c-Fos-like immunoreactivity induced by spreading depression in anaesthetized rats were mediated via the effects of Ca^2+^-entry blockade, which may include an increase in CBF and the prevention of excessive Ca^2+^ influx into brain cells [17]. These results provide the possibility that lomerizine may potentiate CBF and inhibit cortical spreading depression in migraine [17]. Other animal experiments suggested therapeutic effects of lomerizine on CBF. Lomerizine had a greater effect on CBF than on blood pressure and heart rates in anaesthetized rats and beagle dogs [18]. This drug is reported to inhibit voltage-dependent Ca^2+^ channels and 5-hydroxytryptamine (5-HT)_2A_ receptors, leading to suppression of 5-HT-induced contraction in rat basilar artery [19]. Recent study has disclosed that lomerizine recovered visual function in an experimental animal model of optic nerve injury [20]. Therefore, these experimental profiles supported that lomerizine could be clinically effective in cerebral circulatory disturbances, such as migraine status. We first highlighted therapeutic effects of lomerizine on rCBF in a migraineur with aura. This antimigraine drug can regulate rCBF during the interictal phase in migraineurs. Further SPECT studies with numerous migraineurs are needed to elucidate the precise prophylactic mechanism of lomerizine.

## 4. Conclusions

After lomerizine administration had improved MA or visual aura in our patient, brain SPECT revealed restoration of decreased CBFs during the interictal period. Clinicoradiological features of our patients indicated that antimigraine mechanism of lomerizine could contribute to alleviation of interictal cerebral hypoperfusion.

## Figures and Tables

**Table 1:** Changes of regional cerebral blood flows before and after lomerizine administration.

	Before lomerizine		After lomerizine	
	Right	Left	Right	Left
Callosomarginal region	47.3	47.4	59.0	59.4
Precentral region	44.8	50.7	59.4	60.0
Central region	45.2	47.6	67.7	63.4
Parietal region	41.7	43.8	61.0	57.6
Angular region	44.3	46.7	59.4	60.0
Temporal region	48.1	50.6	52.4	51.9
Posterior region	48.8	44.2	58.7	58.6
Pericallosal region	53.0	54.1	59.8	59.9
Lenticular nucleus	53.6	52.8	51.1	49.0
Thalamus	48.2	53.1	49.2	51.7
Hippocampus	46.0	42.2	45.1	43.7
Cerebellar hemisphere	58.5	59.2	57.9	57.4

Data were shown in mL/100 g/min.

## References

[1] Olesen J, Larsen B, Lauritzen M (1981). Focal hyperemia followed by spreading oligemia and impaired activation of rCBF in classic migraine. *Annals of Neurology*.

[2] Lauritzen M, Olesen J (1984). Regional cerebral blood flow during migraine attacks by Xenon-133 inhalation and emission tomography. *Brain*.

[3] Olesen J, Friberg L, Olsen TS (1990). Timing and topography of cerebral blood flow, aura, and headache during migraine attacks. *Annals of Neurology*.

[4] Merckx E, Schiepers C, Verbruggen A, Janssens J, De Roo M (1994). Brain perfusion scintigraphy with ^99m^Tc ethylcysteinate dimer for migraine accompagnee. *Clinical Nuclear Medicine*.

[5] Seto H, Shimizu M, Futatsuya R (1994). Basilar artery migraine reversible ischemia demonstrated by Tc-99m HMPAO brain SPECT. *Clinical Nuclear Medicine*.

[6] Ferrari MD, Haan J, Blokland JAK (1995). Cerebral blood flow during migraine attacks without aura and effect of sumatriptan. *Archives of Neurology*.

[7] Soriani S, Feggi L, Battistella PA, Arnaldi C, De Carlo L, Stipa S (1997). Interictal and ictal phase study with Tc 99m HMPAO brain SPECT in juvenile migraine with aura. *Headache*.

[8] La Spina I, Vignati A, Porazzi D (1997). Basilar artery migraine: transcranial doppler EEG and SPECT from the aura phase to the end. *Headache*.

[9] Iwamoto T, Morita T, Kanazawa T, Ohtaka H, Ito K (1988). Effects of KB-2697, a new calcium antagonist, and other diphenylpiperazines on [3H]nitrendipine binding. *Japanese Journal of Pharmacology*.

[10] Gotoh F, Tashiro K, Kutsuzawa N (1995). Clinical evaluation of KB-2796 (lomerizine hydrochloride) on migraine: late phase II study. *Clinical Evaluation*.

[11] Gotoh F, Fukuuchi Y, Tashiro K (1995). Clinical evaluation of lomerizine on migraine: double-blind study in comparison with demetotiazine. *Clinical Evaluation*.

[12] Gotoh F, Fukuuchi Y, Tashiro K (1995). Long-term trial of lomerizine hydrochloride for migraine. *Japanese Pharmacology and Therapeutics*.

[13] Imai N, Konishi T, Serizawa M, Okabe T (2007). Do the effects of long-term lomerizine administration differ with age?. *Internal Medicine*.

[14] Takeuchi R, Yonekura Y, Matsuda H, Konishi J (2002). Usefulness of a three-dimensional stereotaxic ROI template on anatomically standardised ^99m^Tc-ECD SPET. *European Journal of Nuclear Medicine*.

[15] Matsuda H, Yagishita A, Tsuji S, Hisada K (1995). A quantitative approach to technetium-99m ethyl cysteinate dimer: a comparison with technetium-99m hexamethylpropylene amine oxime. *European Journal of Nuclear Medicine*.

[16] Köseoglu E, Talaslioglu A, Gönül AS, Kula M (2008). The effects of magnesium prophylaxis in migraine without aura. *Magnesium Research*.

[17] Shimazawa M, Hara H, Watano T, Sukamoto T (1995). Effects of Ca channel blockers on cortical hypoperfusion and expression of c-Fos-like immunoreactivity after cortical spreading depression in rats. *British Journal of Pharmacology*.

[18] Hara H, Shimazawa M, Sasaoka M (1999). Selective effects of lomerizine, a novel diphenylmethylpiperazine Ca^2+^ channel blocker, on cerebral blood flow in rats and dogs. *Clinical and Experimental Pharmacology and Physiology*.

[19] Ishii M, Kobayashi S, Ohkura M, Yamamoto R, Shimizu S, Kiuchi Y (2009). Inhibitory effect of lomerizine, a prophylactic drug for migraines, on serotonin-induced contraction of the basilar artery. *Journal of Pharmacological Sciences*.

[20] Selt M, Bartlett CA, Harvey AR, Dunlop SA, Fitzgerald M (2010). Limited restoration of visual function after partial optic nerve injury; a time course study using the calcium channel blocker lomerizine. *Brain Research Bulletin*.

